# Conservation of Ancient Genetic Pathways for Intracellular Persistence Among Animal Pathogenic Bordetellae

**DOI:** 10.3389/fmicb.2019.02839

**Published:** 2019-12-11

**Authors:** Israel Rivera, Bodo Linz, Kalyan K. Dewan, Longhuan Ma, Christopher A. Rice, Dennis E. Kyle, Eric T. Harvill

**Affiliations:** ^1^Department of Infectious Diseases, College of Veterinary Medicine, University of Georgia, Athens, GA, United States; ^2^Center for Tropical and Emerging Global Diseases, University of Georgia, Athens, GA, United States; ^3^Department of Cellular Biology, University of Georgia, Athens, GA, United States

**Keywords:** *Bordetella*, evolution, intracellular survival, macrophages, transcriptomics, stress response and adaptation

## Abstract

Animal and human pathogens of the genus *Bordetella* are not commonly considered to be intracellular pathogens, although members of the closely related classical bordetellae are known to enter and persist within macrophages *in vitro* and have anecdotally been reported to be intracellular in clinical samples. *B. bronchiseptica*, the species closest to the ancestral lineage of the classical bordetellae, infects a wide range of mammals but is known to have an alternate life cycle, persisting, replicating and disseminating with amoeba. These observations give rise to the hypothesis that the ability for intracellular survival has an ancestral origin and is common among animal-pathogenic and environmental *Bordetella* species. Here we analyzed the survival of *B. bronchiseptica* and defined its transcriptional response to internalization by murine macrophage-like cell line RAW 264.7. Although the majority of the bacteria were killed and digested by the macrophages, a consistent fraction survived and persisted inside the phagocytes. Internalization prompted the activation of a prominent stress response characterized by upregulation of genes involved in DNA repair, oxidative stress response, pH homeostasis, chaperone functions, and activation of specific metabolic pathways. Cross species genome comparisons revealed that most of these upregulated genes are highly conserved among both the classical and non-classical *Bordetella* species. The diverse *Bordetella* species also shared the ability to survive inside RAW 264.7 cells, with the single exception being the bird pathogen *B. avium*, which has lost several of those genes. Knock-out mutations in genes expressed intracellularly resulted in decreased persistence inside the phagocytic cells, emphasizing the importance of these genes in this environment. These data show that the ability to persist inside macrophage-like RAW 264.7 cells is shared among nearly all *Bordetella* species, suggesting that resisting phagocytes may be an ancient mechanism that precedes speciation in the genus and may have facilitated the adaptation of *Bordetella* species from environmental bacteria to mammalian respiratory pathogens.

## Introduction

Three closely related species of the gram-negative bacterial genus *Bordetella* make up the group of respiratory pathogens known as the classical bordetellae. These include the notorious human pathogen *Bordetella pertussis*, which is the etiological agent of pertussis or whooping cough (Mattoo and Cherry, 2005) and the closely related *B. parapertussis*, a species which consists of two distinct lineages that cause pertussis-like disease in humans and pneumonia in sheep, respectively (Porter et al., 1994). The third species, *B. bronchiseptica*, infects a wide range of mammals including many domesticated animals (Goodnow, 1980), causing a variety of pathologies ranging from chronic asymptomatic infection to acute bronchopneumonia. Multi locus sequence typing and genome comparisons revealed that *B. pertussis* and *B. parapertussis* independently arose from a *B. bronchiseptica*-like ancestor (Parkhill et al., 2003; Diavatopoulos et al., 2005). The classical bordetellae possess several partially characterized virulence mechanisms (Skarlupka et al., 2019) that are studied in the context of what is viewed as a completely extracellular life cycle in their mammalian hosts (Melvin et al., 2014). Yet, *in vitro* experiments convincingly demonstrated that the classical bordetellae can survive intracellularly within mammalian phagocytic cells (Banemann and Gross, 1997; Lamberti et al., 2010; Gorgojo et al., 2012), an ability that appears to have descended from ancestral progenitor species that lived in the environment (Hamidou Soumana et al., 2017) and acquired the ability to resist phagocytic killing by amoebae that are ubiquitous environmental predators (Taylor-Mulneix et al., 2017b). In fact, *B. bronchiseptica*, the species that most closely resembles the environmental ancestor of the classical bordetellae, can survive within amoeba and also disperse along with amoebic spores, highlighting a novel strategy for an environmental life cycle (Taylor-Mulneix et al., 2017a). These observations strongly suggest that intracellular survival may be an ancestral trait that might have affected the adaptation of *Bordetella* spp. from environmental bacteria to mammalian respiratory pathogens (Taylor-Mulneix et al., 2017b; Linz et al., 2019).

Despite not being commonly considered an intracellular pathogen, *B. pertussis* has repeatedly been recovered from dendritic cells and alveolar macrophages (Hellwig et al., 1999; Carbonetti et al., 2007; Paddock et al., 2008). These studies showed that *B. pertussis* is able to modulate human macrophages by secreting a wide range of proteins upon entry, which allows them to reside within the host cells. Interestingly, the ability to reside inside macrophages is not unique to *B. pertussis*, as recovery from macrophages have been confirmed for all classical bordetellae, including *B. parapertussis* and *B. bronchiseptica* (Gorgojo et al., 2012; Bendor et al., 2015).

In addition to the closely related classical bordetellae, which share about 99% sequence identity throughout their genomes, several other *Bordetella* species have been identified, collectively referred to as non-classical, that display much broader genetic diversity (Supplementary Figure S1). Of these, *B. avium* and *B. hinzii* cause respiratory infections in poultry and wild birds (Kersters et al., 1984; Vandamme et al., 1995). *B. pseudohinzii* was identified as a pathobiont in several mouse breeding colonies (Ivanov et al., 2015, 2016) and was recently shown to cause chronic ear infection in mice (Dewan et al., 2019). *B. trematum* is an opportunistic human pathogen that can cause severe skin disease and chronic otitis media (Vandamme et al., 1996). *B. petrii* was originally isolated from an anaerobic bioreactor culture enriched from river sediment (von Wintzingerode et al., 2001) and was subsequently isolated from many soil samples (Hamidou Soumana et al., 2017; Garrido-Sanz et al., 2018). Although several genomic features have changed throughout their independent evolution, including acquisition and loss of multiple virulence-associated genes (Linz et al., 2016, 2019), these *Bordetella* species share many characteristics that make them successful animal pathogens.

Since many of the non-classical bordetellae are animal pathogens too, we hypothesized that intracellular survival, the ability to resist digestion by phagocytic cells, may constitute an ancient environmental defense mechanism that facilitated the adaptation of *Bordetella* species to animals. If this were the case, then the ability to survive intracellularly would be expected to be widespread among both classical and non-classical bordetellae with shared, conserved genetic pathways. To test this hypothesis, we analyzed the transcriptome of *B. bronchiseptica* following internalization by macrophages and identified the induced key genes and pathways. Cross species genome comparisons revealed that most of the upregulated genes are highly conserved among the *Bordetella* genus. In agreement, both the classical and non-classical *Bordetella* species have retained the ability to survive inside murine macrophages. The only exception, *B. avium* – a species that has been found only among birds – has lost several of those genes and has lost the ability to survive within macrophages. Deletion of these genes in *B. bronchiseptica* substantially decreased its intracellular survival. These data indicate that the ability to resist phagocytic killing by host macrophages is widespread among the animal pathogenic *Bordetella* species and may have been an important step enabling the evolution of *Bordetella* species as animal pathogens.

## Materials and Methods

### Bacterial Strains and Growth

*Bordetella bronchiseptica* strain RB50, *B. pseudohinzii* 8-296-03, *B. hinzii* L60, *B. petrii* DSM12804, *B. avium* 197N and *B. trematum* H044680328 were grown and maintained on BG agar (Difco) supplemented with 10% defibrinated sheep’s blood (Hema Resources). Liquid cultures were grown overnight at 37°C to mid-log phase (OD ∼0.6) in Stainer Scholte (SS) liquid broth (Stainer and Scholte, 1970). *Klebsiella aerogenes* was grown and maintained on Luria-Bertani (LB) agar (Difco) and liquid cultures were grown at 37°C to mid-log phase in LB broth (Difco).

### Intracellular Bacterial Assays

RAW 264.7 macrophages cells were grown to 80% confluency (∼1 × 10^5^ CFU/well) in Dulbecco’s Modified Eagle Media (DMEM) supplemented with 10% FBS, glucose and glutamine in 48-well tissue-culture plates at 37°C. Bacteria were added in 10 μl PBS containing 10^7^ CFU (MOI of 100), 10^6^ CFU (MOI of 10) or 10^5^ CFU (MOI of 1) as indicated. Plates were centrifuged at 250 *g* for 5 min at room temperature and incubated at 37°C for 1 h, after which gentamicin solution (Sigma-Aldrich) was added to a final concentration of 300 μg/ml. Plates were incubated at 37°C for an additional 1, 3, 7, or 23 h and subsequently washed with PBS. 0.1% Triton-X solution was administered, followed by 5 min incubation and vigorous pipetting to lyse the macrophages. The samples were serially diluted and plated on BG agar plates to quantify total bacteria numbers.

### Electron Microscopy

RAW 264.7 macrophages were seeded in 6-well tissue-culture plates at a density of 1.5 × 10^5^ cells/ml, inoculated with *B. bronchiseptica* RB50 at a MOI of 10:1 and centrifuged for 5 min at 250 *g*. Following 1 h incubation at 37°C, the macrophages were washed with PBS, and DMEM media containing 300 μg/ml gentamicin was added. After 1 h, the macrophages were washed with PBS and suspended in a final volume of 300 μl of PBS. The macrophages were then collected by centrifugation and fixed with fresh 2% glutaraldehyde for Transmission Electron Microscopy at the University of Georgia Electron Microscopy Core Facility.

### Confocal Fluorescent Microscopy

Green fluorescent protein (GFP)-expressing *B. bronchiseptica* strain RB50 (Taylor-Mulneix et al., 2017a) was exposed to RAW 264.7 macrophages at a MOI of 100:1 for 1 h, followed by gentamycin treatment for 1 h. Live cell fluorescence microscopy was performed using a Zeiss Axio Obsever.Z1/7 microscope. Imaging was performed at 488 nm for GFP (green), and transmitted light for DIC II (white) at a magnification of 40x using an LD Plan-Neoflaur 40x/0.4 Korr M27 objective.

### Z-Stack Imaging

RAW 264.7 cells were seeded in 6-well tissue-culture-treated plates with coverslips in the bottom at a density of 1.5 × 10^5^ cells/ml in 3 ml and inoculated with *B. bronchiseptica* RB50 at a MOI of 10 (2 × 10^6^ CFU) 12 h later. To synchronize the bacterial exposure to macrophages the plates were centrifuged at 300 *g* for 10 min. After 45 min incubation at 37°C, bacteria in the supernatant were removed by washing the macrophages three times with 1X PBS. The plates were incubated with DMEM medium containing 300 μg/ml gentamicin for 2 h to kill the remaining extracellular bacteria, and then washed 3 times with 3 ml of 1X PBS. Cells were fixed in 4% paraformaldehyde for 10 min at room temperature. The cells were then washed three times with 1X PBS and subsequently permeabilized with 0.1% Triton X-100 in 1X PBS for 20 min at room temperature. Primary antibodies derived from sera of *B. bronchiseptica*-infected mice were added after dilution in 1X PBS containing 2% BSA. After incubation at room temperature for 1 h, the cells were washed 3 times in 1X PBS. Then, the preparation was incubated with secondary donkey anti-mouse antibodies conjugated to FITC and with phalloidin for actin staining for 1 h. After 3 washes in 1X PBS, the coverslips were removed from the plates and fixed on glass slides with mounting medium containing DAPI. The images were taken with a Zeiss LSM 710 Confocal Laser Microscope for Z-stack imaging at 0.5 μm intervals.

### Intracellular Bacterial Assay for Transcriptional Analysis

RAW 264.7 macrophages were seeded in 6-well tissue-culture plates and inoculated with *B. bronchiseptica* RB50 at a MOI of 100:1. A subset of the bacteria was cultured in DMEM medium without macrophages as the negative control. Following 1 h incubation at 37°C, the remaining bacteria in the supernatant were removed by washing the macrophages with PBS and followed by addition of DMEM medium containing 300 μg/ml gentamicin. After 1 h the DMEM was removed, and the macrophages were washed with PBS. The samples were suspended in 1 ml of TRIzol for RNA extraction.

### RNA Isolation and Sequencing

RNA was extracted from RB50 lysates using TRIzol (Ambion) and the Bacterial RNA isolation Kit (Max Bacterial Enhancement Reagent, Ambion) with implemented PureLink DNase treatment (Invitrogen) following the manufacturer’s instructions. RNA quality was assessed using the NanoDrop 2000 (Thermo Scientific) and BioAnalyzer (Agilent). Samples were submitted for Illumina sequencing at the Molecular Research Laboratory in Shallowater, TX, United States. Ribosomal RNA was depleted from each biological replicate (*n* = 3) during preparation of the Illumina sequencing library.

### Bioinformatic Analyses

Quality control of raw reads was performed using FASTQC and TRIMMOMATIC for filtering of low quality reads and trimming of Illumina library adapters. Filtered reads were mapped to *Bordetella bronchiseptica* RB50 genome assembly NC_002927.3 using “Bowtie2.” The resulting output files were used to evaluate differential gene expression between three biological replicates of intracellular *B. bronchiseptica* (*n* = 3) and controls (*n* = 3) using the “EdgeR” package for the statistical environment R distributed within the Bioconductor project.

### Protein Similarity Analysis

Total protein sequences were extracted from the NCBI archive for: *B. bronchiseptica* RB50 (RefSeq assembly accession: GCF_000195675.1), *B. parapertussis* 12822 (GCF_000195695.1), *B. pertussis* Tohama I (CF_000195715.1), *B. hinzii* L60 (GCF_000657715.1), *B. pseudohinzii* 8-296-03 (GCF_000657795.2), *B. avium* 197N (GCF_000070465.1), *B. petrii* DSM12804 (GCF_000067205.1), and *B. trematum* H044680328 (GCF_900078695.1). Similarities between *B. bronchiseptica* proteins and proteins of the non-classical species were calculated in mGenomeSubtractor (Shao et al., 2010) as the *H* value for each protein, defined as *H* = *i x (l_m_/l_q_)*. H is the highest BLASTp identity score (i), multiplied by the ratio of the matching sequence length (*l*_m_) and the query length (*l*_q_). Based on our previous work (Linz et al., 2018), proteins with an *H* value < 0.5 were considered absent. Pairwise tBLASTx genome comparisons in the Artemis Comparison Tool (Carver et al., 2008) validated proteins with values of H > 0.5 as true orthologs.

### Quantitative Real-Time PCR

Real-time PCR analyses were performed on a QuantStudio (Applied Biosystems) using Power SYBR Green PCR Master Mix (Applied Biosystems). Complementary DNA (cDNA) transcript libraries were prepared from biological triplicates of the control and of bacteria incubated with macrophages in DMEM + 10% FBS. Samples were processed for RNA extraction using TRIzol Reagent (Ambion by Life Technologies) and treated with PureLink DNase (Invitrogen). Primers were manually designed and purchased from IDT (Supplementary Table S1). The cycling parameters were as follows: 5-min preincubation at 95°C followed by 40 cycles of a 2-step PCR at 95°C and 60°C. Gene expression was calculated using the ΔΔCt method with expression of the *16S rRNA* used as reference. Data were analyzed using DataAssist version 3.0 (Applied Biosystems).

### Deletion Mutants

The allelic exchange vector pSS4245 (Inatsuka et al., 2010) was used for the generation of deletion mutants. Briefly, ∼1 kb of DNA flanking each end of the target gene was PCR amplified using primers provided in Supplementary Table S2, joined and inserted into the vector by PIPE cloning (Klock and Lesley, 2009). The construct was verified by sequencing, transformed into *E. coli* SM10λ*pir*, and transferred into the parental *B. bronchiseptica* strain RB50 by mating. Colonies containing the integrated plasmid were selected and incubated on BG agar to stimulate allelic exchange by homologous recombination. Emerging colonies were screened by PCR for replacement of the wildtype by the mutant allele and confirmed by Sanger sequencing. *In vitro* growth curves showed that none of the deletion mutants had growth defects compared to the wildtype strain RB50 (data not shown). For complementation, the target gene was cloned into plasmid pBBR1 (Antoine and Locht, 1992).

### Statistical Analysis

The mean ± standard error (error bars in figures) was determined for all appropriate data. Two-tailed, unpaired student’s *t*-tests were used to determine the statistical significance between two normally distributed populations. GraphPad Prism version 6.04 was used to conduct these statistical tests and to generate figures.

## Results

### *B. bronchiseptica* Entry and Persistence in Murine Macrophages

**FIGURE 1:**
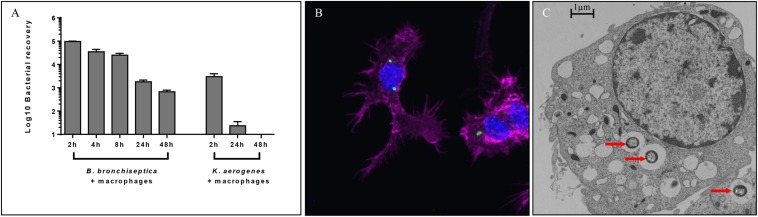
Intracellular survival of *B. bronchiseptica* in murine-derived macrophages. **(A)** Recovery of viable *B. bronchiseptica* RB50 and *K. aerogenes* post internalization by RAW 264.7 macrophages. **(B)** Z-stack fluorescence microscopy localizing *B. bronchiseptica* inside RAW 246.7 cells at 2 h post internalization (p.i.). Purple - F-actin; blue - nucleus; green - *B. bronchiseptica*. **(C)** Transmission electron microscopy of a RAW 264.7 macrophage containing *B. bronchiseptica* RB50 at 2 h p.i. Red arrows depict bacteria in the cell phagosomes. Scale bar: 1 μm.

We had earlier observed using gentamicin protection assays that the prototype *B. bronchiseptica* strain RB50 (*Bb*) can enter and survive within murine macrophage-like cell line RAW 264.7 *in vitro* (Bendor et al., 2015). To determine the number and proportion of bacteria entering these macrophages, we performed an assay of macrophages infected with *Bb* at multiplicities of infection (MOI) of 100, 10 and 1 for 1 h. The percentage of recovery ranged from 0.7 to 1% of the original inoculum at all three MOIs, indicating that a relatively constant fraction of bacteria entered and resisted digestion by macrophages (Figure 1A and Supplementary Figure S2). The observation that the ratio of bacteria to macrophage did not affect survival rate suggested that this is not simply macrophages being overwhelmed or overcome by bacterial numbers. Electron microscopy (Figure 1C), confocal microscopy (Supplementary Figure S3A) and z-stack images (Figure 1B and Supplementary Figure S3B) taken after 2 h incubation confirmed the presence of bacteria within phagocytic vacuoles. Once inside the RAW 264.7 cells, bacterial numbers remained relatively stable and decreased only slowly over time. Bacterial CFUs recovered at 4 and 8 h showed no significant change in numbers for any of the MOIs used, and even at 24 and 48 h intracellular *Bb* were recovered in substantial numbers (Figure 1A). In contrast to *Bb*, *Klebsiella aerogenes* (Figure 1A) failed to persist in RAW 264.7 cells and was recovered at numbers over two orders of magnitude lower.

### *B. bronchiseptica* Transcriptional Response to Internalization by Macrophages

We hypothesized that to survive within professional phagocytic cells, *Bb* would require distinct groups of genes to be transcriptionally modulated once the bacteria reached the intracellular niche. To examine this transcriptional response, we analyzed the RNA profile of intracellular *Bb* at 2 h post inoculation and compared it to that of bacteria grown *in vitro*. Total RNA was isolated from samples collected after antibiotic treatment and sequenced on an Illumina MiSeq (RNA-Seq). On average, 8.8 × 10^5^ reads of intracellular *Bb* (*n* = 3) and 5.6 × 10^6^ reads of the planktonic bacterial control (*n* = 3) mapped to non-rRNA regions of the *Bb* reference genome (NC_002927.3). Those reads were used to evaluate the differential gene expression of *Bb* inside macrophages in comparison to that of bacteria grown *in vitro*.

A Principal Component Analysis (PCA) of the normalized read distribution revealed a clear difference in the global gene expression between intracellular and *in vitro* grown *Bb* (Supplementary Figure S4). The PCA plot showed clustering of the replicates and separation of the two groups along the first principal component (PC1), indicating a distinct transcriptional response to the intracellular environment. Differentially expressed genes that displayed a log2-fold change of either ≥ 1.5 (upregulated genes) or ≤−1.5 (downregulated genes) with a *p*-value < 0.05 were selected for further analysis, which resulted in a list of 318 upregulated and 243 downregulated genes. To validate our RNA-seq dataset we performed a quantitative real-time (qRT) PCR to assess the transcriptional changes in five highly upregulated and four strongly downregulated genes (Supplementary Figure S5), which confirmed the RNA-Seq data.

#### Upregulated Genes

Functional analysis of the transcriptionally upregulated genes showed major changes at the functional levels of metabolic process (102 genes), of cellular process (116 genes), regulation (24 genes) and response to stimuli (8 genes). Gene ontology evaluation revealed enrichment for genes whose products are involved in cellular processes (113 genes) including: DNA repair, protein folding and repair, oxidative stress response, and pH homeostasis, as well as enrichment for metabolic processes (102 genes) such as nutrient assimilation (24 genes) (Table 1). A list of all 318 upregulated genes can be found in Supplementary File S1.

**TABLE 1:** Upregulated genes in intracellular *B. bronchiseptica*.

**Locus_tag**	**Gene**	**logFC**	***P*-value**	**Description**
**DNA repair**				
BB0180	*dksA*	3.8	2.4E-12	RNA polymerase-binding transcription factor
BB1919	*dnaB*	1.4	5.4E-08	Replicative DNA helicase
BB2076	*recA*	1.8	2.1E-08	Recombinase RecA
BB2935	*dps*	2.0	2.0E-07	Putative DNA-binding protein
**Oxidative stress and pH homeostasis**				
BB0020	*risA*	2.1	4.3E-06	Transcriptional regulator RisA
BB1837	*rpoE*	3.2	1.7E-12	RNA polymerase sigma factor RpoE
BB2275	*iscR*	4.2	1.7E-12	Transcriptional regulator IscR
BB2276	*iscS*	2.5	3.3E-10	Cysteine desulfurase
BB2279	*hscB*	1.6	1.5E-07	HscB chaperone
BB2281	*fdx*	1.6	5.5E-04	Ferredoxin, 2Fe–2S
BB3080	*slyA*	3.0	7.2E-06	Transcriptional regulator SlyA
BB3766	*msrP*	1.6	8.7E-08	Protein-methionine-sulfoxide reductase
BB3800	*msrB*	2.1	1.9E-07	Peptide methionine sulfoxide reductase
BB4506	*rpoN*	3.4	9.5E-12	Sigma(54) modulation protein RpoN
BB3942	*fur*	2.5	3.3E-08	Ferric uptake regulator
BB4835	*rpoH*	4.5	2.4E-12	RNA polymerase sigma factor RpoH
**Protein folding**				
BB0178	*hslU*	4.1	2.3E-12	ATP-dependent protease
BB0179	*hslV*	6.0	1.9E-12	ATP-dependent protease
BB0295	*secB*	1.6	1.3E-07	Protein-export protein
BB0501	*htpG*	4.2	2.8E-10	Chaperone protein
BB0962	*groEL*	4.4	8.4E-11	60 kDa chaperonin
BB0963	*groES*	7.8	6.7E-14	10 kDa chaperonin
BB2256		2.4	8.4E-11	ATP-dependent protease
BB3170	*hfq*	2.4	3.0E-07	RNA-binding protein
BB3293	*clpB*	2.6	5.5E-10	Chaperone protein
BB3933	*dnaJ*	1.7	1.1E-07	Chaperone protein
BB3934	*dnaK*	3.9	9.7E-11	Chaperone protein
BB3936	*grpE*	3.9	2.6E-10	Chaperone protein
**Metabolism**				
BB0095	*glcB*	1.9	4.2E-06	Malate synthase G
BB0096	*glcC*	1.9	1.5E-06	Malate synthase G transcriptional regulator
BB3682	*sdhC*	2.3	1.0E-09	Succinate dehydrogenase cytochrome B
BB3684	*mdh*	2.8	8.9E-10	Malate dehydrogenase
BB1850	*acnB*	1.4	1.04.E-7	Aconitate hydratase B
BB4150		2.4	1.5E-07	Putative short-chain dehydrogenase
BB3474	*ompA*	3.1	3.9E-11	Outer membrane protein A
BB3759	*plsX*	1.6	1.9E-09	Phosphate acyltransferase
BB3771	*pagL*	2.4	1.3E-08	Lipid A deacylase
BB0233		2.9	2.3E-09	Putative AMP-binding enzyme
BB1556		4.1	2.4E-12	ABC transporter, ATP-binding protein
BB4592		4.1	1.7E-12	Putative binding-protein-dependent transport
BB0097		3.9	6.4E-10	Putative dehydrogenase
BB1446	*carA*	1.5	6.8E-09	Carbamoyl-phosphate synthase
BB0235		5.1	9.5E-12	Probable transporter
BB4355	*argC*	2.6	5.4E-11	*N*-acetyl-gamma-glutamyl-phosphate reductase
BB1986	*argG*	1.5	2.4E-08	Argininosuccinate synthase
BB2000		2.5	1.3E-09	Putative aldolase
BB3179	*ndk*	2.3	1.9E-09	Nucleoside diphosphate kinase
BB2607	*pyrH*	1.8	2.0E-09	Uridylate kinase
BB3468	*cmk*	1.5	2.8E-08	Cytidylate kinase
BB4376	*nrdA*	2.3	1.6E-09	Ribonucleoside-diphosphate reductase
**Virulence**				
BB2992	*fimA*	2.6	9.7E-11	Fimbrial protein
BB2994	*bvgA*	2.6	2.6E-09	Transcriptional regulator of virulence
BB3424		1.7	3.7E-07	Fimbrial protein
BB3674	*fim2*	6.7	1.7E-12	Serotype 2 fimbrial subunit

*logFC, log2 fold change.*

Expression of *recA*, *dnaB, dps*, and *dksA*, all implicated in the activation of the SOS response and DNA repair (Bearson et al., 1997; Simmons et al., 2008; Lund et al., 2014), was upregulated upon internalization by macrophages. Likewise, genes for protein folding and recycling such as molecular chaperones *groES*, *groEL*, and *htpG*, and protease genes *hslV* and *hslU*, were highly upregulated, as was expression of several other osmotic and heat shock response genes, including *clpB*, *grpE*, *dnaK*, and *dnaJ*.

Congruent to previous studies in other bacterial species (Buchmeier et al., 1997; Zimna et al., 2001; Clements et al., 2002; Gilberthorpe et al., 2007; Chiang and Schellhorn, 2012; Fang et al., 2016), expression of genes that promote resistance against oxidative stress and low pH was upregulated intracellularly, including transcription factor *iscR* and adjacent genes *iscS*, *hscB*, and *fdx*, and the transcription regulators *slyA*, *risA*, and *fur*. Additionally, RNA polymerase sigma factor genes *rpoH*, *rpoN*, and *rpoE* were highly upregulated (Laskos et al., 2004; Delory et al., 2006; Hanawa et al., 2013), as was expression of the RNA chaperone gene *hfq*, which is known to increase resistance against killing by macrophages (Bibova et al., 2013).

Increased transcription of glyoxylate cycle genes such as *mdh*, *sdhC*, *glcB, glcC*, and *acnB*, as well as of numerous ribosomal protein genes implies extensive metabolic activity in the bacterial cell in response to internalization by macrophages. Several genes of fatty acid synthesis pathways such as 3-oxoacyl-ACP reductase BB4150, long chain fatty acid Co-A ligase BB0233, outer membrane protein *ompA*, and ABC transport protein encoded by BB1556, were found to be strongly induced, suggesting increased membrane biosynthesis. Also, we observed an increase in expression of genes involved in amino acid biosynthesis and transport, including BB4592, *carA*, *argC*, and *argG*, and of *de novo* nucleotide biosynthesis (*ndk*, *pyrH*, *cmk*, and *nrdA*).

The fimbria encoding genes *fim2*, *fimA* and BB3424 were the only genes encoding virulence-associated factors among the 318 transcriptionally upregulated genes.

#### Downregulated Genes

The *cya* genes encoding the adenylate cyclase toxin were among the most downregulated genes in our dataset with a log2fold change of about -3 (Table 2 and Supplementary File S1), and expression of the dermonecrotic toxin gene *dnt* was also downregulated. In agreement with previous studies (Hausman et al., 1996; Antoine et al., 2000; Hausman and Burns, 2000), expression of the pertussis toxin operon and the associated type IV secretion system (T4SS) was barely detectable under either condition. Similarly, expression of the type III secretion system (T3SS) encoded by the *bsc* locus was strongly suppressed, resulting in decreased expression of both the apparatus-related and the secretion-related components (Table 2 and Supplementary File S1). In addition to toxins and secretion systems, expression of the O-Antigen–encoding *wbm* locus (*wbmO* – *bplJ*), was significantly downregulated.

**TABLE 2:** Downregulated genes in intracellular *B. bronchiseptica.*

**Locus_tag**	**Gene**	**LogFC**	***P*-value**	**Description**
**O-Antigen**				
BB0130	*wbmO*	−1.8	4.6E-06	O-Antigen biosynthesis protein
BB0138	*wbmG*	−2.2	1.1E-05	Nucleotide sugar epimerase/dehydratase
BB0139	*wbmF*	−1.8	9.4E-06	Nucleotide sugar epimerase/dehydratase
BB0145	*bplL*	−1.8	3.2E-06	Lipopolysaccharide biosynthesis protein
BB0146	*bplJ*	−1.6	1.2E-05	Membrane protein
**Adenylate cyclase toxin**				
BB0325	*cyaB*	−2.9	3.3E-09	Cyclolysin secretion ATP-binding protein
BB0326	*cyaD*	−3.4	1.0E-08	Membrane fusion protein (MFP) family protein
BB0327	*cyaE*	−3.3	5.8E-10	Protein CyaE
BB0328	*cyaX*	−3.9	1.1E-08	Adenylate cyclase transcriptional regulator
**Type 3 secretion system**				
BB1609	*bscF*	−2.1	6.5E-06	Putative type III secretion protein
BB1623	*bcr4*	−2.6	6.9E-08	Uncharacterized protein
BB1624	*bscI*	−2.0	1.3E-04	Putative type III secretion protein
BB1625	*bscJ*	−2.3	4.4E-07	Lipoprotein
BB1627	*bscL*	−2.5	1.1E-04	Type III secretion protein
BB1628	*bscN*	−1.9	5.3E-06	Type III secretion ATP synthase
BB1630	*bscP*	−2.8	4.8E-07	Type III secretion protein
BB1631	*bscQ*	−3.0	4.3E-08	Type III secretion protein
BB1632	*bscR*	−2.1	6.5E-05	Type III secretion protein
BB1634	*bscT*	−3.0	3.0E-06	Type III secretion protein
BB1635	*bscU*	−2.8	7.9E-07	Type III secretion protein
BB1636	*bscW*	−2.8	9.9E-05	Type III secretion protein
BB1637	*bscC*	−1.9	3.9E-07	Type III secretion protein
**Electron transport**				
BB3827		−4.6	6.0E-10	Putative membrane protein
BB3828	*nuoN*	−3.0	6.9E-10	NADH-quinone oxidoreductase subunit N
BB3829	*nuoM*	−2.3	2.5E-08	NADH-ubiquinone oxidoreductase, chain M
BB3830	*nuoL*	−2.4	6.9E-10	NADH-ubiquinone oxidoreductase, chain L
BB3834	*nuoH*	−1.7	1.3E-07	NADH-quinone oxidoreductase subunit H
BB3835	*nuoG*	−1.9	3.7E-08	NADH-quinone oxidoreductase
BB3836	*nuoF*	−2.0	5.7E-09	NADH-quinone oxidoreductase subunit F
**Cell division**				
BB4188		−2.2	2.4E-08	Uncharacterized protein
BB4193	*ftsZ*	−2.5	4.3E-10	Cell division protein FtsZ
BB4194	*ftsA*	−3.4	3.3E-09	Cell division protein FtsA
BB4195	*ftsQ*	−3.4	6.0E-09	Cell division protein FtsQ
BB4196	*ddl*	−3.5	2.1E-07	D-alanine–D-alanine ligase
BB4197	*murC*	−3.0	1.1E-08	UDP-*N*-acetylmuramate–L-alanine ligase
BB4198	*murG*	−3.6	8.1E-10	Undecaprenyl-PP-MurNAc-pentapeptide-UDPGlcNAc GlcNAc transferase
BB4199	*ftsW*	−3.6	3.6E-08	Cell division protein FtsW
BB4200	*murD*	−3.0	5.2E-09	UDP-*N*-acetylmuramoyl-L-alanyl-D-glutamate synthetase
BB4201	*mraY*	−2.8	1.3E-08	Phospho-*N*-acetylmuramoyl-pentapeptide-transferase
BB4202	*murE*	−2.1	1.0E-09	Multifunctional fusion protein

*logFC, log2 fold change.*

Notably, several genes with important functions in cell structure biogenesis and proliferation were also downregulated inside macrophages, including cell division genes *ftsZ*, *ftsA*, and *ftsQ* and cell wall synthesis genes *murC*, *murG*, *ftsW*, and *murD*. Similarly, expression of a large gene locus (BB3827 to BB3836) encoding the oxidative respiratory chain was significantly downregulated, including NADH dehydrogenase genes *nuoN*, *nuoM*, *nuoL*, and *nuoH*.

Taken together, *B. bronchiseptica* responded to internalization by macrophages by rapid changes in its transcriptional profile, that were marked by suppression of growth and virulence, and strong activation of the bacterial stress response, including DNA and protein repair and pH homeostasis, and suppression of cell division, putative virulence factors and oxidative respiration.

### The Non-classical Bordetellae and Intracellular Persistence

**FIGURE 2:**
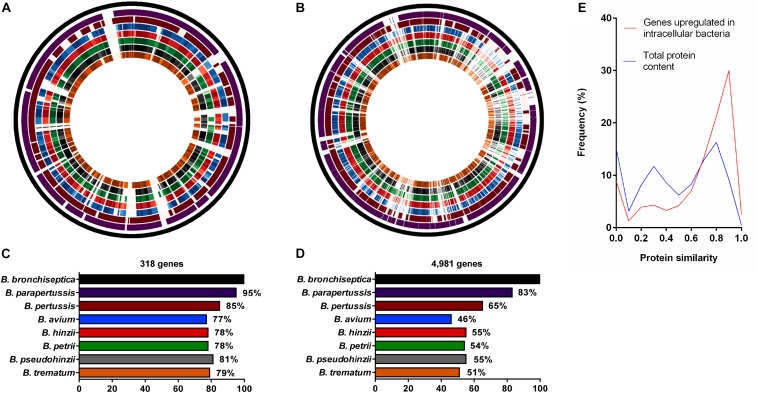
Comparative analysis of genes upregulated during intracellular survival and their presence/absence in non-classical *Bordetella* species. Analysis of protein similarity of **(A)** 318 *B. bronchiseptica* genes upregulated in macrophages and **(B)** 4,981 genes in the entire genome of *B. bronchiseptica* strain RB50 in comparison to the non-classical *Bordetella* species. From outside to inside: Circle 1: Virtual genome of *B. bronchiseptica* strain RB50. Circles 2–8: Visual representation of protein similarity between *B. bronchiseptica* RB50 and classical (circles 2–3) and the non-classical species (circles 4–8) represented as color shades with darker shades indicating higher protein similarity. **(C)** 77–81% of the genes upregulated in intracellular *B. bronchiseptica* were conserved among the non-classical species, **(D)** in contrast to only 46–55% of the 4,981 *B. bronchiseptica* genes in the entire genome. **(E)** Line plot showing the frequency of protein similarities.

Since the human-restricted pathogens *B. pertussis* and *B. parapertussis* arose from *B. bronchiseptica*-like ancestors and can persist inside human macrophages, we evaluated whether the 318 upregulated genes were conserved among the three classical *Bordetella* species. Two hundred and seventy two intact genes (86%) were identified in the genome of *B. pertussis* strain Tohama I, the other genes were missing or truncated by frameshifts or premature stop codons. Similarly, 301 of the 318 genes (95%) were present in the genome of *B. parapertussis* strain 12822, showing conservation of most genes (Figures 2A,C).

While many non-classical bordetellae are also human and animal pathogens, their ability to persist inside phagocytic cells has not been evaluated. Therefore, we tested the presence or absence of the upregulated genes among the non-classical *Bordetella* species, including the bird pathogens *B. hinzii* (Vandamme et al., 1995) and *B. avium* (Kersters et al., 1984), the mouse pathogen *B. pseudohinzii* (Ivanov et al., 2016), the human opportunistic pathogen *B. trematum* (Vandamme et al., 1996), and the environmental species *B. petrii* (von Wintzingerode et al., 2001). We calculated the protein similarity (*H* value) of the 318 genes upregulated in *B. bronchiseptica* inside macrophages and their corresponding homologs in the non-classical bordetellae, with a gene considered to be present with a protein similarity value of *H* ≥ 0.5. An average of 77–81% of the 318 upregulated genes were present in the non-classical species (Figures 2A,C) with 95 (30%) of the genes displaying similarity values of *H* ≥ 0.9 (Figure 2E). In contrast, only 46–55% of the total of 4,981 evaluated *B. bronchiseptica* genes were identified in the genomes of the non-classical species (*P* < 0.0001), where only 448 (9%) of the genes reached protein similarity scores of *H* ≥ 0.9 (Figures 2B,D,E).

**FIGURE 3:**
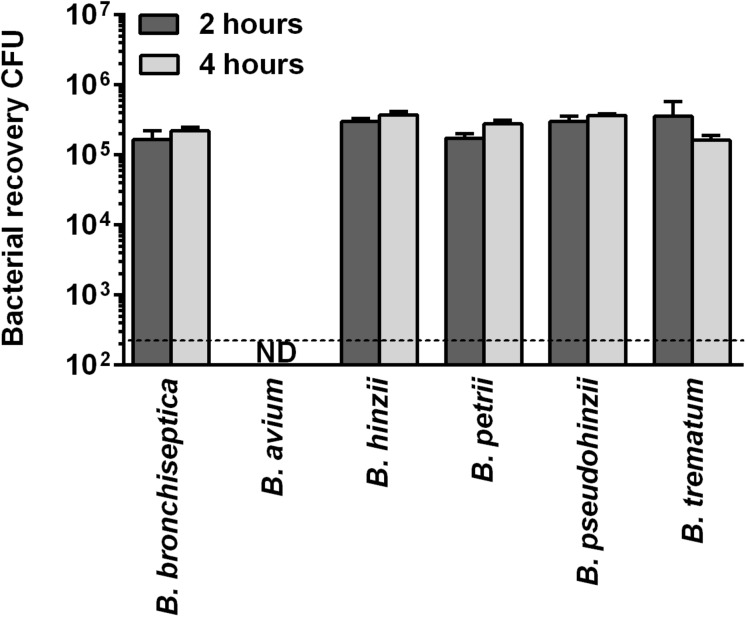
Intracellular survival of non-classical bordetellae within macrophages. The non-classical bordetellae were recovered from macrophages in numbers similar to *B. bronchiseptica*. The only exception, *B. avium*, failed to survive internalization by macrophages.

This high evolutionary conservation of genes that are upregulated in *B. bronchiseptica* during intracellular survival in phagocytic cells suggests that the non-classical bordetellae may be able to persist inside macrophages. To test this hypothesis, the non-classical species were assessed for intracellular survival in RAW 264.7 macrophages for 2 and 4 h. All examined *Bordetella* species were recovered, with the exception of *B. avium* (Figure 3). The inoculated *B. pseudohinzii*, *B. hinzii*, *B. trematum* and *B. petrii* bacteria survived internalization by macrophages at similar rates to *B. bronchiseptica*. The genomes of these species share 222 out of the 318 (70%) transcriptionally upregulated genes, which implies a critical function for intracellular persistence in mammalian phagocytic cells.

**FIGURE 4:**
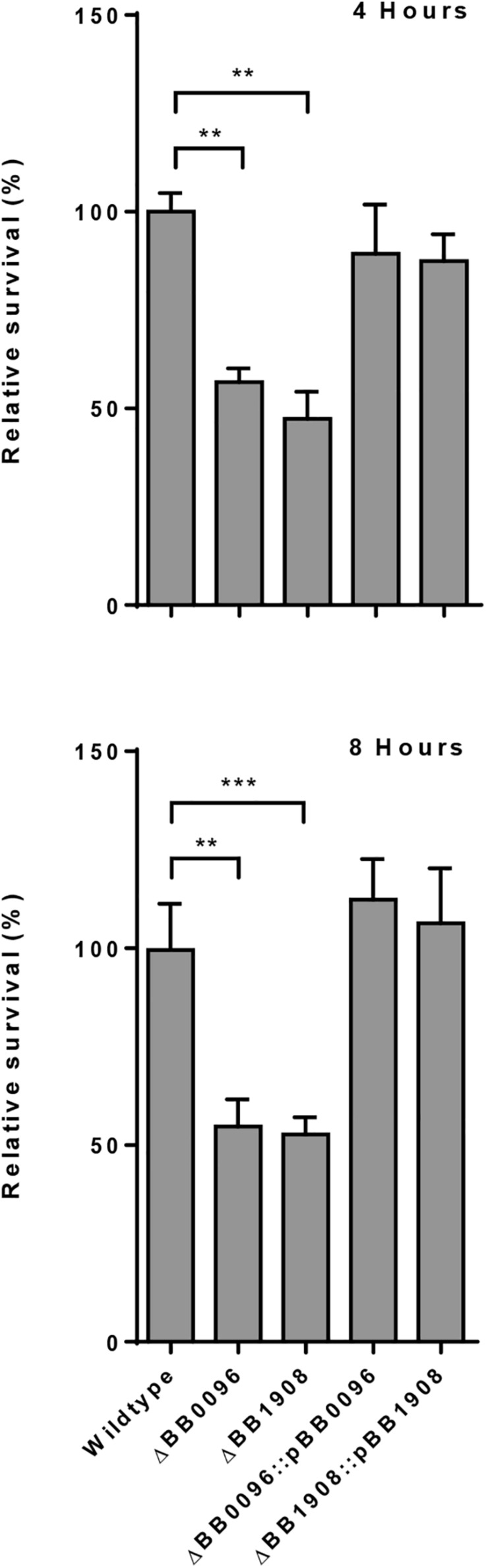
Assessment of *B. bronchiseptica* deletion mutants for intracellular survival. Deletion of malate synthase regulator gene BB0096 or tripartite tricarboxylate transporter gene BB1908 resulted in significantly reduced bacterial recovery. Plasmid-borne complementation of the gene deletion restored the wildtype phenotype. ^∗∗^*p* < 0.01; ^∗∗∗^*p* < 0.001.

In contrast to the other analyzed *Bordetella* species, *B. avium* was severely impaired in its ability to persist inside macrophages. Only 0.001% of the inoculum was recovered after 2 h and no viable bacteria were detected after 4 h. Therefore, we performed a comparative genome analysis to identify transcriptionally upregulated genes that were only missing in *B. avium*, which resulted in the identification of six genes (Table 3). Deletion of two of these genes (BB0096 and BB1908) resulted in a significant reduction in intracellular survival (Figure 4 and Supplementary Figure S6). Complementation of these knock-out mutants with plasmid-borne gene copies restored the wildtype phenotype in both mutants (Figure 4), confirming that loss of malate synthase transcriptional regulator *glcC* (BB0096) or the tripartite tricarboxylate transporter BB1908 negatively impacts intracellular persistence in macrophages. In addition, previous studies showed important roles of transcriptionally upregulated (Table 1) *risA* and *hfq* genes in intracellular persistence of *Bb* and *B. pertussis* (Zimna et al., 2001; Bibova et al., 2013). We also assessed *Bb* knock-out mutants of several other transcriptionally upregulated genes (Supplementary Table S3), however, intracellular survival of none of the tested mutants was significantly different from the RB50 wildtype strain.

**TABLE 3:** Genes upregulated in intracellular *B. bronchiseptica* and absent in *B. avium.*

**Locus_tag/gene**	**logFC**	**Protein**
BB0096/*glcC*	1.9	Malate synthase transcriptional regulator
BB1908	1.6	Tripartite tricarboxylate transporter receptor
BB1948	1.9	Glutamate transport periplasmic receptor
BB1999	2.5	Tripartite tricarboxylate transporter receptor
BB2944	1.6	LysR-family transcriptional regulator
BB4150	2.4	3-ketoacyl-(acyl-carrier-protein) reductase

*logFC, log2 fold change.*

## Discussion

Most bacterial pathogens have specialized to either an intracellular or extracellular lifestyle, which determines the focus in studies on bacterial pathogenesis. The classical species of the genus *Bordetella* are broadly known as extracellular pathogens. However, an increasing number of publications have reported recovery of viable bacteria from phagocytic host cells *in vitro*, providing evidence for at least transient intracellular survival or persistence. There are also anecdotal reports of clinical samples harboring intracellular *B. pertussis* leading to speculation on the significance of this intracellular bacterial population in pathogenicity (Higgs et al., 2012).

Here we showed that intracellular survival and persistence is not restricted to the three classical bordetellae, but that the non-classical species *B. hinzii*, *B. pseudohinzii*, *B. trematum*, and *B. petrii* survived at equally high proportions, establishing the ability to survive internalization by phagocytic host cells as a common feature among the animal pathogenic bordetellae. Considering that the environmental species *B. petrii* did not differ from the animal pathogenic species, this common genotypic and phenotypic trait suggests the ability to survive predation by eukaryotic phagocytic cell precedes speciation in the genus.

A common ability suggests an ancestral origin and a common set of genes that are required for intracellular survival. Indeed, of the 318 *B. bronchiseptica* genes found to be transcriptionally upregulated during intracellular exposure, about 80% were present in the genomes of non-classical *Bordetella*, with 222 genes (70%) shared between all species (Figure 2A). The only exception was the bird pathogen *B. avium*, which was missing six out of these 222 genes and failed to persist inside macrophages (Table 3). Interestingly, *B. avium* has one of the smallest genomes in the *Bordetella* genus with only 3.73 Mb in size (Sebaihia et al., 2006) suggesting that it may have undergone genome reduction during its evolution and adaptation to a specific host (Parkhill et al., 2003; Linz et al., 2016; Taylor-Mulneix et al., 2017b).

**FIGURE 5:**
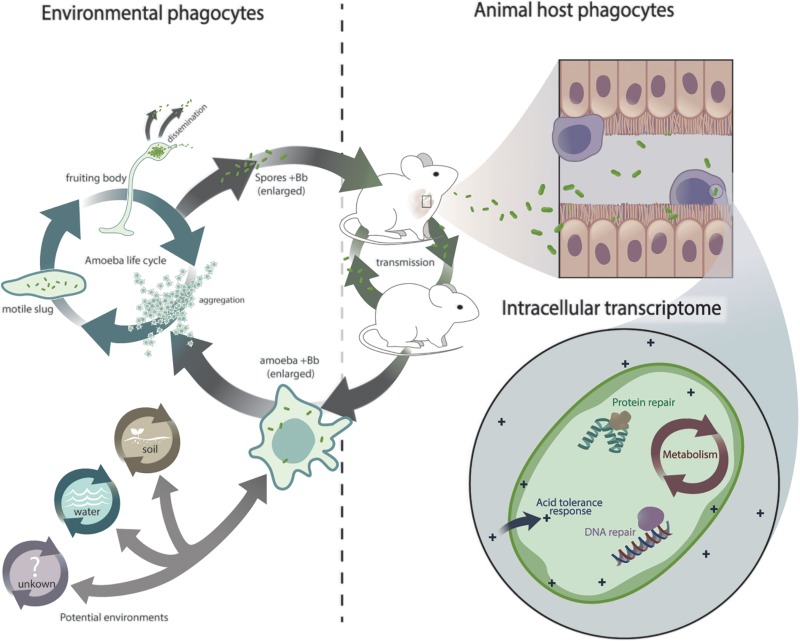
Schematic representation of *Bordetella* exposure to eukaryotic phagocytes and its transcriptional response. Hypothetical scenario depicting *Bordetella* exposure to interconnected lifecycles and adaptation from environmental phagocytes to animal phagocytes.

The apparent localization of the intracellular *B. bronchiseptica* within phagosomes (Figure 1C) suggests that the bacteria are rapidly exposed to a variety of damaging conditions, including low pH, bactericidal factors and resource starvation. To survive such harsh conditions many pathogenic bacteria have evolved mechanisms to maintain cell homeostasis and prevent DNA damage and cell death (Simmons et al., 2008). We observed the transcriptional hallmarks of a general SOS response (Bearson et al., 1997; Lund et al., 2014), characterized by suppression of cell division *via* downregulation of the *fts* locus (Table 2) and by upregulation of DNA repair genes, of protein chaperone genes, and of *B. bronchiseptica* homologs (*rpoH*, *fur*, *risA*) of the *E. coli* acid tolerance genes *rpoS*, *fur* and *phoP* (Table 1 and Figure 5) (Simmons et al., 2008).

Intracellular persistence was also accompanied by metabolic changes (Figure 5 and Tables 1, 2). As expected under micro-aerophilic/hypoxic conditions inside macrophages, expression of the *nuoF* – *nuoN* genes that encode the oxidative respiratory chain was strongly suppressed. In contrast, genes of the glyoxylate/TCA cycle showed elevated expression levels, including malate synthase G gene *glcB* and its transcriptional activator *glcC*, malate dehydrogenase *mdh*, citrate synthase *gltA*, and aconitase *acnB*. The glyoxylate cycle is important in the utilization of acetate or fatty acids as the main carbon source and may be essential to provide hexoses for nucleotide and amino acid biosynthesis under intracellular conditions (Pellicer et al., 1999; Munoz-Elias and McKinney, 2006). *B. bronchiseptica* genes involved in biosynthesis of nucleotides, amino acids and fatty acids were indeed upregulated, consistent with limited access to these molecules. The absence of the malate transcriptional activator *glcC* and tricarboxylic transporter BB1908 may explain the failure of *B. avium* to persist inside macrophages.

Many intracellular pathogens such as *Burkholderia pseudomallei* employ protein secretion systems to facilitate replication and spread inside their hosts (Stevens et al., 2002). Interestingly, our assays were conducted at 37°C, a temperature known to induce phosphorylation of *bvgA*, which in turn induces expression of virulence factors (Prugnola et al., 1995). Yet under these intracellular conditions *B. bronchiseptica* displayed strong suppression of other known virulence factors, including the operons encoding both T3SS and the adenylate cyclase toxin (ACT) expression, modification and secretion. While stress conditions such as low pH have been reported to induce the expression of virulence factors in many pathogenic bacteria (Rathman et al., 1996; Bearson et al., 1997), the suppression of virulence in *B. bronchiseptica* occurred despite their intracellular vacuolar location where similar low pH environments are expected. We had earlier reported that the avirulent stage is required for survival, persistence and replication of *B. bronchiseptica* within amoeba (Taylor-Mulneix et al., 2017a), which strongly suggests that repression of virulence within the intracellular environment is part of an ancient conserved stress response in the genus.

Taken together, our results show that upon internalization by macrophages a certain proportion of bordetellae are killed, but thousands of bacteria can adapt and modulate gene expression to cope with this new environment. Rapid transcriptional adaptation was marked by what can be considered a general stress response against professional phagocytes that included increased expression of genes involved in DNA and protein repair, acid tolerance and metabolism (Figure 5). Conservation of these genes throughout the genus and the demonstrated ability of non-classical species, including the environmental *B. petrii*, to persist inside macrophages suggests that this response to phagocytes is not confined to the commonly studied classical bordetellae. It appears to represent an ancient pathway that preceded speciation in the genus and thus likely arose from a common ancestor. The two independent but interconnected transmission cycles of *B. bronchiseptica* in environmental amebae and in mammalian hosts (Figure 5) lead us to speculate that early interaction with these environmental phagocytes may have played a role in the origin of this response, which subsequently facilitated the adaptation to higher animals and thus the evolution of *Bordetella* from environmental microbes to animal and human pathogens.

## Data Availability Statement

The raw data supporting the conclusions of this article will be made available by the authors, without undue reservation, to any qualified researcher.

## Author Contributions

IR, BL, and EH conceived the study. IR, BL, KD, LM, CR, DK, and EH designed the experiments. IR, BL, KD, LM, and CR performed the experiments. IR, BL, KD, LM, CR, and EH analyzed the data. IR, BL, KD, and EH wrote the manuscript.

## Conflict of Interest

The authors declare that the research was conducted in the absence of any commercial or financial relationships that could be construed as a potential conflict of interest.
