# Supplementary material for: Conservation of Ancient Genetic Pathways for Intracellular Persistence Among Animal Pathogenic Bordetellae
Source: Front Microbiol. 2019 Dec 11;10:2839. doi: 10.3389/fmicb.2019.02839 (PMC6917644; doi:10.3389/fmicb.2019.02839)
Supplement: Supplementary file 1 [file Data_Sheet_1.PDF]

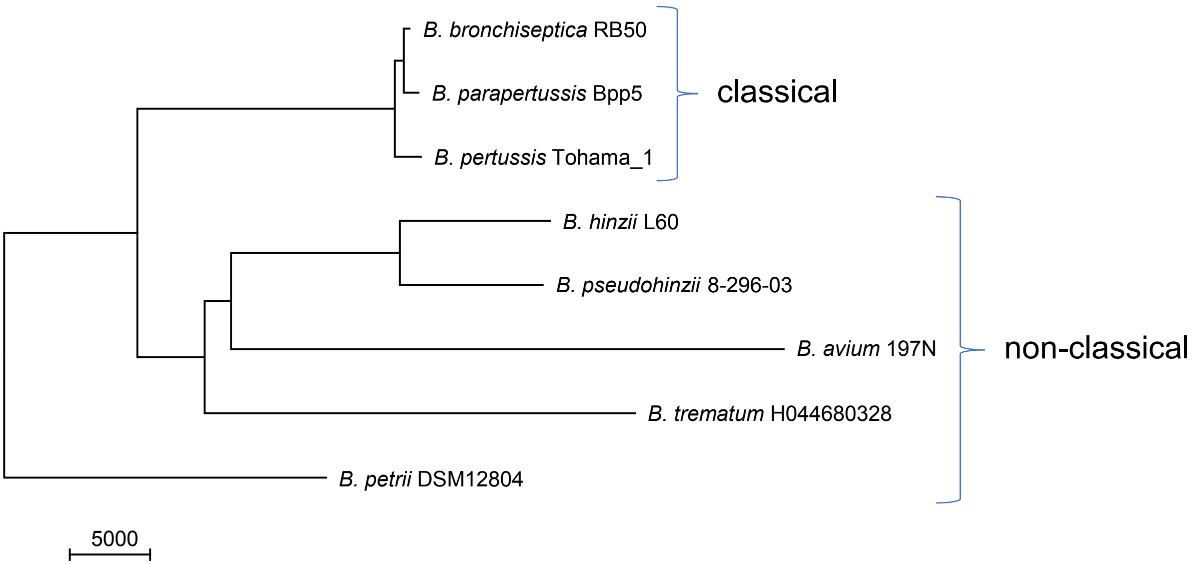


**Figure S1. Genome-wide SNP-based phylogeny of *Bordetella* species.**
The genomes of the eight analyzed species formed three phylogenetic clades, similar to Linz et al., 2016. The phylogeny was based on 373,499 base pairs shared between all eight genomes. Genes with more than one copy per genome such as *16S rRNA* were not included. Scale bar: number of differences.

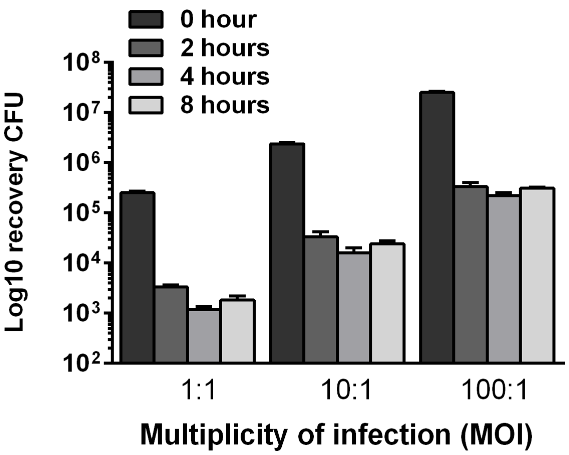


**Figure S2. Intracellular survival of *B. bronchiseptica* strain RB50 in macrophages at several time points post inoculation with a multiplicity of infection (MOI) of 1:1, 10:1 or 100:1.** Bacteria were consistently recovered at 0.7% to 1.0% of the inoculum at each of the used MOIs.

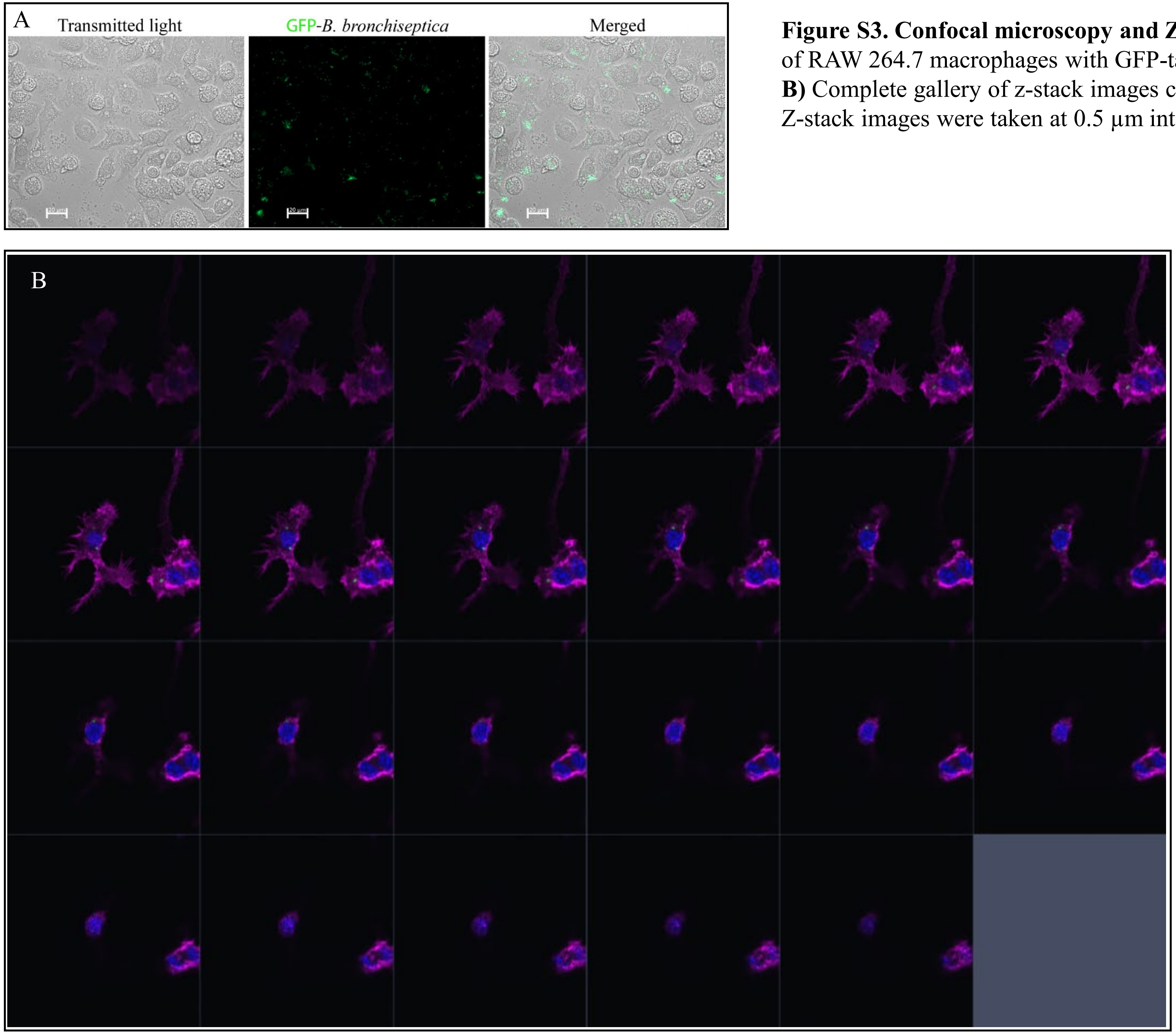


**Figure S3. Confocal microscopy and Z-stack gallery images. A)** Confocal fluorescent microscopy of RAW 264.7 macrophages with GFP-tagged *B. bronchiseptica* RB50 2 hours p.i. Scale bar: 20 µm. **B)** Complete gallery of z-stack images confirming intracellular localization of *B. bronchiseptica* RB50. Z-stack images were taken at 0.5 µm intervals. purple – F-actin; blue – nucleus; green – *B. bronchiseptica*.

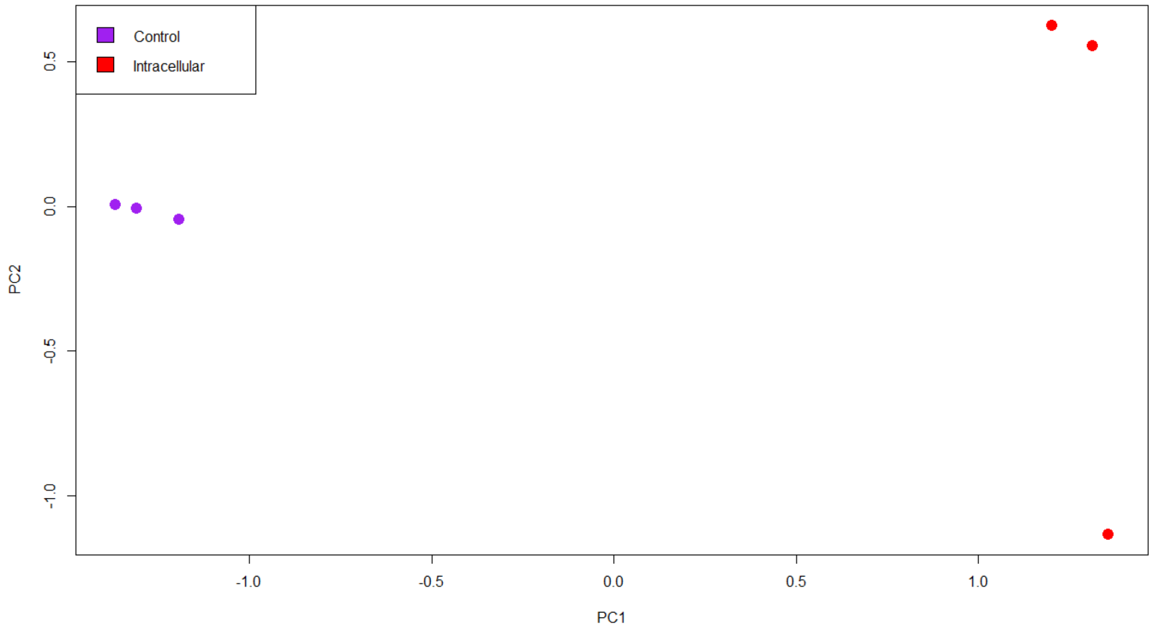


**Figure S4. Principal component analysis.** Principal-component (PC) analysis of normalized transcript abundance in intracellular *B. bronchiseptica* RB50 incubated with macrophages for 2 hours compared to RB50 cultured in DMEM + 10% FBS medium as the control.

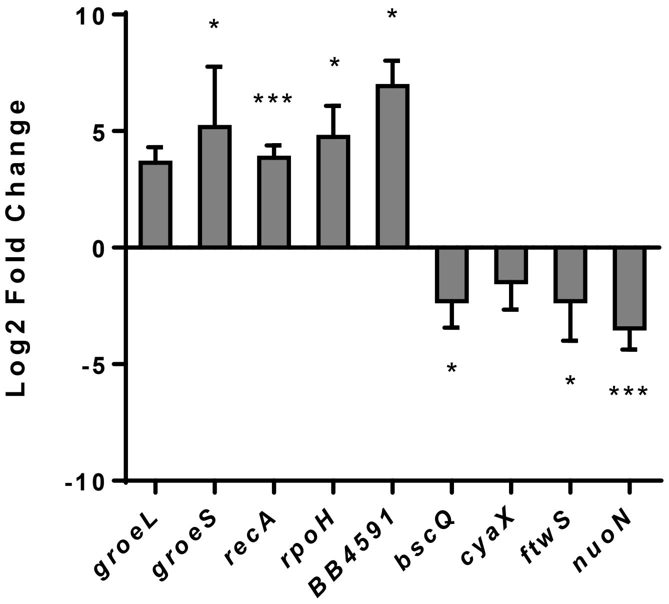


**Figure S5. Verification of *B. bronchiseptica* gene expression inside RAW 264.7 macrophages by qRT PCR**. Expression of 9 genes identified in the transcription data displayed as the mean 2-fold change in gene expression levels (with standard deviations) of *B. bronchiseptica* RB50 isolated from macrophages at 2 hours p.i. relative to RB50 cultured in DMEM + 10% FBS medium. Values are based on 3 independent experiments. Asterisks indicate significantly different expression levels; * $p \leq 0.05$; *** $p < 0.001$).

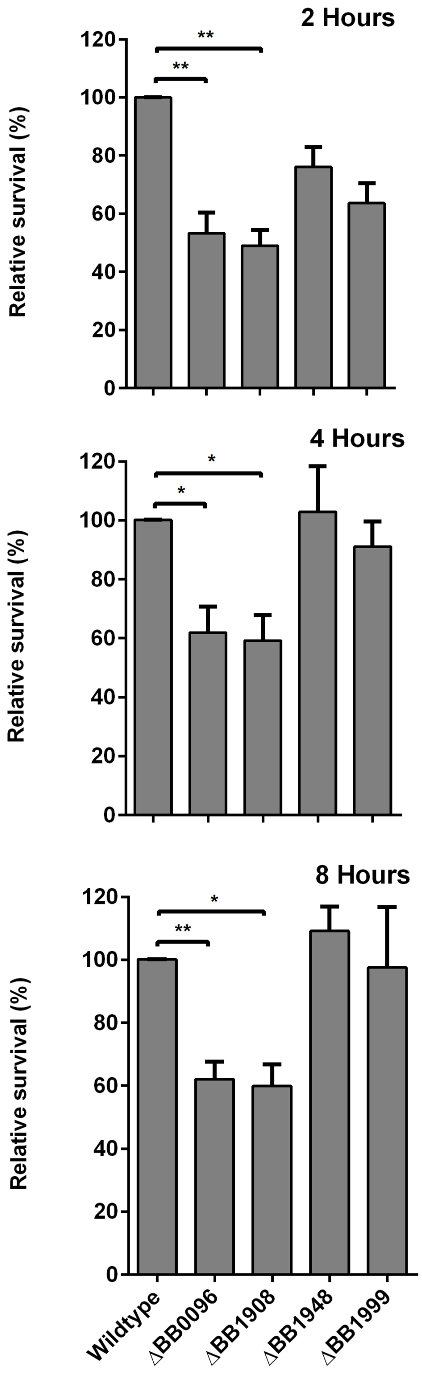


**Figure S6. Assessment of *B. bronchiseptica* deletion mutants for intracellular survival.** Deletion of malate synthase regulator gene BB0096 or tripartite tricarboxylate transporter gene BB1908 resulted in significantly reduced bacterial recovery at 2, 4, and 8 hours post inoculation. In contrast, the knock-out mutants of genes BB1948 and BB1999 did not display significant differences in comparison to wildtype bacteria.

**Table S1. Primers for quantitative real-time PCR.**

| **Gene** | **Direction** | **Sequence (5' → 3')** | **Bp** | **Tm** |
|---|---|---|---|---|
| *groeL* | Forward | GACGATCCGTATGTCCTGATCTA | 23 | 63.0°C |
| | Reverse | CGGATGTTGTTCACGACCAG | 20 | 63.0°C |
| *groeS* | Forward | GAAGACCGAAGACGGCAAGAT | 21 | 64.0°C |
| | Reverse | CTGGATCACGGCGAGGATTT | 20 | 64.0°C |
| *recA* | Forward | CAAGCTGACGGCCACCAT | 18 | 58.5°C |
| | Reverse | GCACCGAGGAATAGAACTTGAG | 22 | 55.3°C |
| *rpoH* | Forward | CGCAAGCTGTTCTTCAACCT | 20 | 63.0°C |
| | Reverse | TCATCGTCGTCCTGGCTTTC | 20 | 64.0°C |
| BB4591 | Forward | ACCTGACCCTTAGCCACAAC | 20 | 64.0°C |
| | Reverse | TTCGTCGATGAAGGCGAACA | 20 | 64.0°C |
| *bscQ* | Forward | TACATCGGCCTGACGGTTC | 19 | 64.0°C |
| | Reverse | CATGCCTCGACAGACATCCT | 20 | 64.0°C |
| *cyaX* | Forward | GCTCGATGCGCAGAGTTAT | 19 | 62.0°C |
| | Reverse | CGCATACGACACATAGGGATAG | 22 | 62.0°C |
| *ftwS* | Forward | GGCATCAACGGCAAGTATTTC | 21 | 62.0°C |
| | Reverse | AATGCGACAACTGGTAGGC | 19 | 62.0°C |
| *nuoN* | Forward | CATCGTCCAGACCAACTTCAA | 21 | 62.0°C |
| | Reverse | CGTAGGTCAGCATGTAGAACAG | 22 | 62.0°C |
| *16S rRNA* | Forward | GGATTAGATACCCTGGTA | 18 | 46.0°C |
| | Reverse | CCGTCAATTCCTTTGAGTTT | 20 | 50.9°C |

**Table S2. Primers for generation of gene knock-out mutants**

| Gene | Sequence (5' → 3') |
|---|---|
| BB0096-UF | CCTATGCTAGGGCGGCCGCACTAGGGTCGATCTGGATTTCG AAGTGCAGGC |
| BB0096-intUR | GAGCACGCCATGGTACGACCGTAGCCCGTCGACGCCTGTTT CCATGACTTG |
| BB0096-intDF | CAAGTCATGGAAACAGGCGTCGACGGGCTACGGTCGTACCA TGGCGTGCTC |
| BB0096-DR | GCAGGTCCGGATCTGTACACCTAGGAGCTGCGCGAGTTCGA CCTGCAGC |
| BB1908-UF | CCTATGCTAGGGCGGCCGCACTAGTCTGTGGATCGGCTTGC TTGGATGAG |
| BB1908-intUR | GGCTTATTGCAGAACGATGTTGGCCTGGAGCAGTTGCAAAC CTTTGCGTAGCAT |
| BB1908-intDF | ATGCTACGCAAAGGTTTGCAACTGCTCCAGGCCAACATCGT TCTGCAATAAGCC |
| BB1908-DR | GCAGGTCCGGATCTGTACACCTAGGGGATGGATCAGGTAGA TGCCGAACGTGAGC |
| BB1948-UF | CCTATGCTAGGGCGGCCGCACTAGTCGAGCAGTATTCGGAA TCGTCGTCGTCTTCCCAGT |
| BB1948-intUR | CGCTTAGTCGATCTTCACGTTGGCTTCACGACGTTGCATGGT TTCCCCTGTAGGAAG |
| BB1948-intDF | CTTCCTACAGGGGAAACCATGCAACGTCGTGAAGCCAACGT GAAGATCGACTAAGCG |
| BB1948-DR | GCAGGTCCGGATCTGTACACCTAGGGCCTATGTACTTCTTGA CGGTCTCTGGCGTTGC |
| BB1999-UF | CCTATGCTAGGGCGGCCGCACTAGGTCGTTCGATAACGCCG TG |
| BB1999-intUR | TGGCCTGCTTGAAGACCAACCTGAAGCCGATCTTCG |
| BB1999-intDF | ATCGGCTTCAGGTTGGTCTTCAAGCAGGCCACGTTC |
| BB1999-DR | GCAGGTCCGGATCTGTACACCTAGGGCCTGCATCTGCTCGA AGGC |
| pSS4245-Forward | CTAGTGCGGCCGCCCTAGCATAGG |
| pSS4245-Reverse | CCTAGGTGTACAGATCCGGACCTGC |

Red: primer region complementary to pSS4245

Blue: primer region complementary to the corresponding intUR/intDF primer

**Table S3. Intracellular survival of *B. bronchiseptica* RB50 gene knock-out mutants.**

| | | | | 2 hours | | 4 hours | | 8 hours | |
|---|---|---|---|---|---|---|---|---|---|
| **Locus_tag** | **Gene** | **Product** | **Fold Change** | **CFU** | **P-value** | **CFU** | **P-value** | **CFU** | **P-value** |
| BB0096 | *glcC* | Malate synthase transcriptional regulator | 1.92 | 8.6E+04 | **0.00** | 6.3E+04 | **0.04** | 7.4E+04 | **0.01** |
| BB0235-BB0229 | | Branched-chain amino acid transport system | 2.91 | 1.6E+05 | 0.71 | 1.1E+05 | 0.26 | 9.8E+04 | 0.29 |
| BB1908 | | Tripartite tricarboxylate transporter receptor | 1.56 | 8.3E+04 | **0.00** | 6.3E+04 | **0.05** | 7.5E+04 | **0.02** |
| BB1948 | | Glutamate transport periplasmic receptor | 1.92 | 1.2E+05 | 0.14 | 1.0E+05 | 0.58 | 1.3E+05 | 0.14 |
| BB1999-BB2000 | | Aldolase; Tripartite tricarboxylate transporter receptor | 1.91 | 1.1E+05 | 0.07 | 1.0E+05 | 0.57 | 1.3E+05 | 0.52 |
| BB3674-BB3676 | *fim2* | Serotype 2 fimbrial subunit precursor | 6.71 | 1.3E+05 | 0.33 | 1.5E+05 | 0.00 | 9.1E+04 | 0.10 |
| BB4590-BB4593 | | ABC transporter | 4.0-7.7 | 1.4E+05 | 0.54 | 1.6E+05 | 0.00 | 1.1E+05 | 0.88 |
| RB50 | | | NA | 1.5E+05 | NA | 9.3E+04 | NA | 1.1E+05 | NA |

P-values of significantly lower intracellular survival in comparison to wildtype *B. bronchiseptica* RB50 in **bold**.
